# PRISM: An open source framework for the interactive design of GPU volume rendering shaders

**DOI:** 10.1371/journal.pone.0193636

**Published:** 2018-03-13

**Authors:** Simon Drouin, D. Louis Collins

**Affiliations:** McConnell Brain Imaging Center, Montreal Neurological Institute and Hospital McGill University, McGill University, Montreal, Quebec, Canada; Bangladesh University of Engineering and Technology, BANGLADESH

## Abstract

Direct volume rendering has become an essential tool to explore and analyse 3D medical images. Despite several advances in the field, it remains a challenge to produce an image that highlights the anatomy of interest, avoids occlusion of important structures, provides an intuitive perception of shape and depth while retaining sufficient contextual information. Although the computer graphics community has proposed several solutions to address specific visualization problems, the medical imaging community still lacks a general volume rendering implementation that can address a wide variety of visualization use cases while avoiding complexity. In this paper, we propose a new open source framework called the Programmable Ray Integration Shading Model, or PRISM, that implements a complete GPU ray-casting solution where critical parts of the ray integration algorithm can be replaced to produce new volume rendering effects. A graphical user interface allows clinical users to easily experiment with pre-existing rendering effect building blocks drawn from an open database. For programmers, the interface enables real-time editing of the code inside the blocks. We show that in its default mode, the PRISM framework produces images very similar to those produced by a widely-adopted direct volume rendering implementation in VTK at comparable frame rates. More importantly, we demonstrate the flexibility of the framework by showing how several volume rendering techniques can be implemented in PRISM with no more than a few lines of code. Finally, we demonstrate the simplicity of our system in a usability study with 5 medical imaging expert subjects who have none or little experience with volume rendering. The PRISM framework has the potential to greatly accelerate development of volume rendering for medical applications by promoting sharing and enabling faster development iterations and easier collaboration between engineers and clinical personnel.

## Background

### Introduction

In recent years, the ubiquity of programmable Graphics Processing Units (GPUs) has enabled the use of direct volume rendering (DVR) to visualize 3D medical images at interactive frame rates. An important advantage of DVR is that it enables interactive exploration of complex 3D volumes, such as the ones produced by magnetic resonance (MR) and computed tomography (CT) scanners, without the need for prior segmentation and processing. For this reason, DVR has the potential to greatly improve the visualization capabilities of a wide variety of medical imaging devices such as surgical navigation systems, interventional radiology systems and picture archiving and communication systems (PACS).

Despite the interactivity of current DVR techniques, producing an informative and perceptually sound picture for every visualization task remains a challenge. The simple color and opacity transfer functions implemented in most DVR systems lack the discriminative power to separate different tissue types with similar volume intensities. It is often difficult to adjust the rendering parameters to provide a correct perception of shape and depth of anatomical structures. In many cases, rendered images are overly cluttered and present inconvenient occlusion patterns where structures of interest are hidden by less relevant parts of the anatomy. There is an extensive body of literature that addresses one or more of these problems. A family of techniques, often referred to as *focus+context*, seek to emphasize important image structures (focus) and filter out of less relevant details while maintaining sufficient information for orientation (context) [1–7]. Different types of transfer functions enable better separation of tissue types [8,9] and several techniques improve shape and depth perception using lighting effects [10–20] or depth-based strategies [21–25]. In many cases, simple heuristics combined with information from different image modalities can greatly improve the quality of rendering for specific medical tasks[26–29]. Unfortunately, only a few of these techniques have found their way to systems that are commonly used in clinical research and medical practice.

In this paper, we present the Programmable Ray Integration Shading Model, or PRISM. The goal of the PRISM framework is to implement a GPU-based DVR module that can produce a wide variety of volume rendering effects while remaining extremely simple to use. We achieve this goal by allowing users to modify key areas of the rendering pipeline in real time. Clinically oriented personnel or researchers are able to experiment with interchangeable blocks available from a database of examples presented in this paper. For more technically oriented researchers and programmers, PRISM enables real-time editing of those blocks of code to produce novel rendering effects. Rather than defining a new GPU volume rendering method, the main contribution of this paper is to propose a framework that exposes an abstract representation of the volume rendering pipeline while hiding complex details of the technical implementation. We demonstrate both the flexibility of the framework for programmers by showing volume rendering effects examples that can be implemented with no more than a few lines of code and the ease of use of the framework through a user study where non-programmers were able to reproduce several effects by combining existing blocks of code through a graphical user interface. The framework is implemented as a set of classes for the Visualization Toolkit (VTK), which is already used as the rendering backend of a majority of open source medical imaging programs such as 3D Slicer[30,31], MITK[32,33], ITKSnap[34,35], CustusX[36,37] and IBIS Neuronav[38,39]. The framework can thus easily be integrated in all these platforms already in use in many clinical research facilities to enable rapid prototyping of new visualization methods aimed at specific clinical applications.

### Volume rendering basics

In DVR, volumes of data sampled on a rectilinear grid such as MR and CT scans are viewed as blocks of semi-transparent medium of varying density. The scalar value of the data at every point within the volume is mapped to optical properties such as color and opacity. Images are rendered by integrating the effect of those optical properties along a viewing ray defined for every pixel.

**Fig 1:**
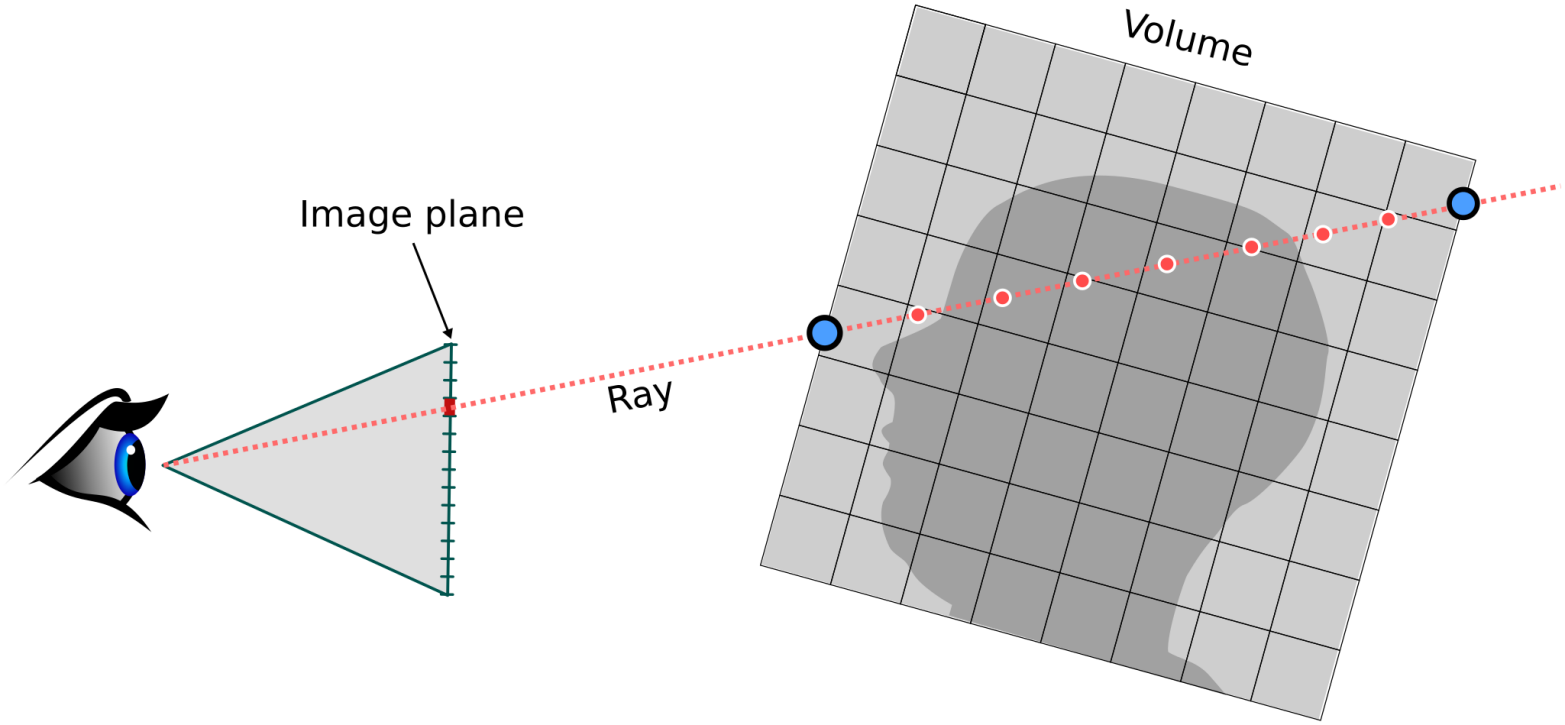
Illustration of the emission-absorption model used to compute the color of a pixel. A ray is cast from the virtual camera through the pixel in the image plane. The intersection with the volume is found and the volume is sampled at regular interval, the sample value is mapped to optical properties which are composited to obtain the pixel color.

In practice, considering every possible emission, reflection and refraction of light in the medium is intractable and approximations must be made. Max *et al*. established a series of optical models[40] that approximate the rendering equation with different levels of realism. The most widely used is the emission-absorption model where each voxel of the volume is treated as a source of light that is partially absorbed by other voxels on the way to a virtual camera. The rendering integral can be approximated by accumulating the emissive contribution of volume samples taken at regular interval along a ray cast from every pixel of the image plane as illustrated in Fig 1.

Several methods have been proposed to accelerate computation of DVR, some of which use the ray-casting approach explicitly and others that use mathematically equivalent formulations to take advantage of different hardware architectures. Lacroute and Levoy reduce the DVR computation to a sequence of simple 2D image composites and a final warping operation[41]. Early approaches to DVR on the GPU relied on 2D Texture mapping[42]. Later, the introduction of 3D texture mapping allowed rendering of a set of view-aligned planes [43], removing some of the artefacts usually associated with 2D texturing methods. The introduction of GPU programming languages has enabled users to implement ray-casting on graphics hardware[44]. The technique has become very popular due to its simplicity and speed through optimization with early ray termination (ERT) and empty space skipping [45]. In this paper, we use the GPU ray-casting approach to volume rendering.

### Related work

The most widely used DVR implementation in the field of medical imaging is the open source Visualization Toolkit’s (VTK) volume rendering module. VTK implements various GPU-based rendering methods including 2D texture mapping (vtkVolumeTextureMapper2D), 3D texture mapping (vtkVolumeTextureMapper3D) and ray casting (vtkVolumeRayCastMapper). The implementation of the ray casting method is similar to the one proposed by Kruger *et al*.[44]. It allows users to choose between different ray casting functions, including compositing, iso-surface and maximum intensity projection, but it can render only a single volume at a time. Krissian *et al*. [46] proposed a VTK class that can simultaneously render 2 volumes and later, Bozorgi *et al*. [47] extended the idea to support an arbitrary number of volumes.

Several other open source volume rendering solutions exist. Many of them rely internally on VTK (e.g., 3D Slicer[30,31], ParaView[48,49], Tomviz[50]). However, some of these alternative solutions are available only as standalone applications, which makes it difficult to incorporate them in new applications. ImageVis3D[51,52] implements the ClearView[1] framework to separate structures of interest from contextual information and supports very high resolution volumes. VolumeShop[53,54] is a Windows-only application that allows for the combination of several volumes, but development has stopped since 2005. Voreen[55,56] supports complex illumination models including shadows and scattering. While development snapshots of the source code are available to download, there is no public code repository. Inviwo[57,58] reproduces all the basic functionality of Voreen, but is more advanced and is openly developed. It is currently the cutting edge platform for research in volume rendering. However, Inviwo aims at a user base of computer graphics researchers. Its interface is complex and the code base is not suitable for integration in a medical imaging application where medical personnel could experiment with new rendering techniques within their typical workflow. The Visualization Library (VL)[59] provides a thin layer of abstraction on top of the graphics API (OpenGL) that facilitates the implementation of volume renderers. This approach maximizes flexibility for expert graphics programmers but cannot be used directly by clinically oriented researchers or medical personnel. MeVisLab[60,61] supports the implementation of modules to create custom applications, but it is not open-source. The core of the framework has extensive DVR capabilities: shadow mapping, boundary and silhouette enhancement, local ambient occlusion and tone shading. Through extensions, it supports a mechanism similar to PRISM to write custom volume rendering shaders[62], but using a slice-based rendering method instead of ray casting.

Unlike these other toolkits and software packages, the PRISM framework presented in this paper can easily be embedded in existing medical imaging application as it is based on a VTK class. The PRISM framework simplifies many aspects of volume rendering and thus enables many different rendering techniques to be implemented with only a few lines of code as shown in the examples below. PRISM is developed in a public Git repository and distributed under the very liberal BSD 3-clause license.

## Methods

The PRISM framework implements a GPU-accelerated ray-casting volume rendering method. The core of the framework is implemented as a VTK class (vtkPRISMVolumeMapper), which enables easy integration in different medical imaging applications and combination with all other types of graphical primitives supported by VTK. Internally, the ray-casting algorithm is implemented as an OpenGL Shading Language (GLSL) program. The IBIS Neuronav system[38], an open source image-guided neurosurgery platform, is the first application to embed the PRISM framework in a graphical user interface (GUI) to facilitate its use by non-programmers.

### VTK class functionality

The basic functionality of the vtkPRISMVolumeMapper class is similar to that of other VTK volume mappers, with the following additional features:

1. **Custom GLSL code**: users can provide snippets of custom GLSL code to replace 3 key steps of the ray casting algorithm. This feature is detailed in the GLSL ray integration section below.
2. **Multiple input volume support**: Users can provide multiple input volumes to the mapper, each of which is associated with a color and opacity transfer function, which may be accessed from the custom GLSL code.

The combination of these additional features greatly facilitates the implementation of a wide variety of volume rendering effects, as we will demonstrate below.

### VTK class implementation

The DVR technique used in vtkPRISMVolumeMapper is similar to the method of Kruger *et al*.[44]. The method consists of two rendering passes. The first pass determines the equation of the rays cast for every pixel in the image and the second pass computes the ray integration.

**Fig 2:**
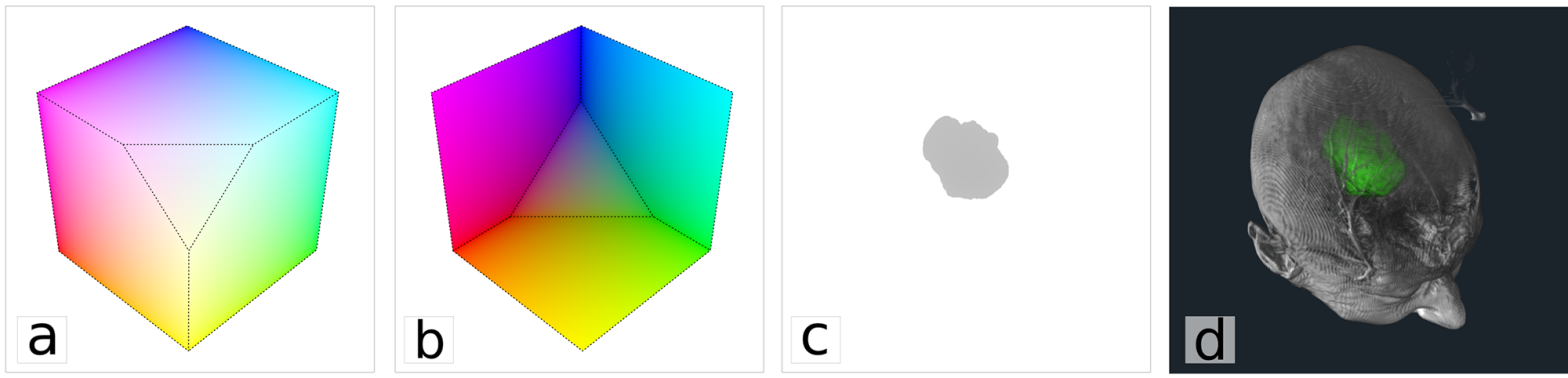
Illustration of the 2-pass volume rendering algorithm implemented in PRISM. In the first pass, the front and back faces of the bounding box of the volume are rendered in two separate textures as illustrated in (a) and (b). The color assigned to the bounding box vertices encodes the normalized coordinate of the vertices in the volume. The box is clipped using near and far clipping planes of the renderer to avoid creating holes in the image. In the second pass, textures (a) and (b) are used to compute ray equation and run the integration. Bounds of the ray are adjusted using depth buffer accumulated by VTK for the rendering of other primitives, in this case, the 3D surface of a tumor (c). Doing so allows the correct interleaving of surfaces and volumes as shown in (d).

The first pass takes advantage of the fast interpolation capabilities of the GPU to compute intersection of rays with the volume for every pixel. The front and back faces of a polygonal model corresponding to the bounding box of the volume are rendered in two separate textures. Each of the corners of the box is assigned a color that encodes the normalized 3D coordinate of the vertex in the volume. Before rendering, the box is clipped with the near and far clipping planes of the renderer to avoid creating holes when those planes intersect the volume (e.g. when the camera is inside of the volume). The resulting textures, shown in Fig 2a and 2b, effectively encode, for each pixel, the two intersection points of a ray with the volume.

The second pass consists of drawing an image-sized plane with the ray integration shader program enabled. For every pixel, the program samples from the front and back face textures rendered in the first pass to obtain the intersection points between the ray and the volume and computes the ray equation. Bounds of the ray integration can further be adjusted by sampling the depth buffer (Fig 2c) generated by other polygonal geometry in the 3D scene. This enables correct rendering of polygonal models that are interleaved with the volumes. Fig 2c shows the content of the depth buffer prior to volume rendering (but after rendering the surface of a tumor using a standard surface rendering technique) and Fig 2d shows an MR volume rendered with PRISM and correctly interleaved with the tumor surface.

### Ray integration

The ray integration method is described in Algorithm 1 below.

**Algorithm 1**. Ray integration algorithm where rayStart and rayEnd are the position, in normalized volume space, of the entry and exit points of the ray, sampleRGBA is the color and alpha value accumulated at each point along the ray and pixelRGBA is the final pixel color. Init, Volume and StopCondition functions in bold characters are placeholders for custom code provided by users.

rayStart = sample (textureFront)

rayEnd = sample (textureBack)

rayDir = normalize (rayEnd−rayStart)

depth = sample (depthBufferTexture)

AdjustRay (rayStart, rayEnd, depth)

**Init (pixelRGBA, rayStart, rayEnd)**

pos = rayStart

pixelRGBA = (0, 0, 0, 0)

while pos < rayEnd

{

 sampleRGBA = (0, 0, 0, 0)

 for each volume v

  sampleRGBA = **Volume(v, pos, sampleRGBA)**

 pixelRGBA = alpha_blend (pixelRGBA, sampleRGBA)

 pos + = rayDir * step

 **StopCondition()**

}

Algorithm 1 is similar to conventional ray integration algorithms such as the one implemented in the VTK volume rendering module, except for the three lines highlighted in bold characters. These functions are placeholders for custom GLSL code provided by the user, which we will refer to as **Init**, **Volume** and **StopCondition** shaders for the remainder of the paper. The **Init** shader enables users to alter start, end and orientation of the rays as well as the initial pixel color before ray integration starts. It can also be used to initialize variables that are used in the **Volume** shaders. The **Volume** shader enables users to have full control over the contribution of each volume to the color accumulated at each step of the ray integration procedure. Finally, the **StopCondition** shader is used to stop ray integration before the volume is completely traversed, and is thus used mostly for optimization purposes. To facilitate the development of such custom shaders, the PRISM framework provides a set of built-in variables and functions. Built-in variables include properties of the volume, the current ray and the rendering environment such as the position of the camera and light source. Built-in functions facilitate sampling from the volumes, converting and evaluating transfer functions. Built-in functions also exist to help with lighting calculation, for example, the ComputeGradient function, which computes the intensity gradient of the volume at a specific location using the finite difference method, can be used to approximate surface normal. Built-in function are provided only to facilitate the development of shaders, but users are free to provide their own implementation of a similar functionality. A detailed list of built-in function and variables is provided in S1 Appendix.

Note that because the custom GLSL code is provided to vtkPRISMVolumeMapper as strings, applications using the class can change the code without recompiling, enabling users to experiment with new shaders interactively.

### Integration in IBIS Neuronav

To facilitate real time editing of ray casting shaders, it is convenient to embed the vtkPRISMVolumeMapper class in a graphical user interface (GUI). Currently, only one such implementation exists as part of the IBIS Neuronav platform[38] where PRISM is integrated as an IBIS plugin. The plugin enables users to combine an arbitrary number of volumes, interactively manipulate the parameters of the mapper and edit transfer functions for each of the volumes. Most importantly, the GUI permits editing of the custom shader code and the results are shown in real-time in the 3D window of the application.

### Volume rendering effects database

The integration of the PRISM framework in a GUI such as the IBIS platform enables the rapid development of new volume rendering effects. Despite the simplicity of the shading language involved, the shader editing feature of the GUI interface is primarily intended for computer graphics experts and requires a minimum basic knowledge of GLSL programming. For the benefit of a less technical audience, we created the PRISM database, a place where the PRISM volume rendering examples are shared publicly. The database consists of a list of files, each of which loads one volume rendering example into the IBIS Neuronav system. Currently, the database contains the IBIS files required to reproduce all the volume rendering examples discussed in this paper. The database and compiled executables of IBIS Neuronav as well as instructions on how to load the examples in the program are available at *ibisneuronav*.*org/prism*.

Collection of the data used in the study was approved by the Montreal Neurological Institute's Research Ethics Board (REB). All procedures followed were in accordance with the ethical standards of the responsible committee on human experimentation (institutional and national) and with the Helsinki Declaration of 1975, as revised in 2008[63]. Written informed consent was obtained from all patients for being included in the study.

### Usability study

To demonstrate that the PRISM framework can be useful and simple enough to use for non-programmers and users without a deep understanding of the principles of volume rendering, we designed a user study using the GUI of the PRISM plugin in the IBIS platform. The study consisted of three parts. The first part is a five minutes tutorial given by the experimenter that summarizes the basic principles of volume ray-casting and the functionality of the different GUI elements of the PRISM plugin. In the second part, users sit at a workstation where the IBIS software is installed and are asked to use the GUI interface of the PRISM plugin to reproduce three different volume rendering examples shown on an alternate screen. We use three examples that are described in details in the results section below: volume carving, blood flow and decluttering. For each example, the subjects need to use the interface to find the right combination of volumes, existing shaders and parameters. For the first two examples, a step by step description of the actions to take was provided on a sheet of paper (c.f., S2 Appendix). For the last example, users were required to find the right combination of parameters to produce the desired visualization without explicit instructions. For this last example, a maximum of five minutes was allocated. Throughout the execution of the tasks, users were asked to think aloud, describe their actions and report any inconsistencies or non-intuitive features in the interface. Each session was video-recorded for further analysis. In the final part of the study, users are asked to fill a web-based questionnaire about their experience with PRISM. The questionnaire consists of 3 parts. The first part assesses prior knowledge of volume rendering of the user. The second part is a standard System Usability Scale (SUS) test [64] and finally, the third part consists of questions that are more specific to the PRISM plugin interface. A copy of the questionnaire is provided in the S3 Appendix. All the questions, except for the last one, are answered on a Likert scale from 1 to 5 (1 = strongly disagree, 5 = strongly agree). The last question requested suggestion from the user on possible improvements to make the system more accessible. In total, 5 subjects took part in the study. They are all medical imaging experts who have little or no experience with volume rendering.

## Results

### Performance evaluation

The performance of the PRISM framework is highly dependent upon the implementation of user-specified shaders as well as the number of volumes used for rendering. To verify the base implementation, we compare the default modes of PRISM and the VTK volume renderer (vtkGPUVolumeRaycastMapper). In its default mode, PRISM has only one input volume, no **Init** or **StopCondition** shaders and a simple **Volume** shader that produces a color-mapped volume sample for every step of the ray integration. It is thus equivalent to the existing VTK volume renderer. An interesting optimization that is made possible by the **StopCondition** shader in PRISM is early ray termination (ERT). It consists in stopping ray integration for a pixel after the opacity (alpha) has reached a certain value.

**Fig 3:**
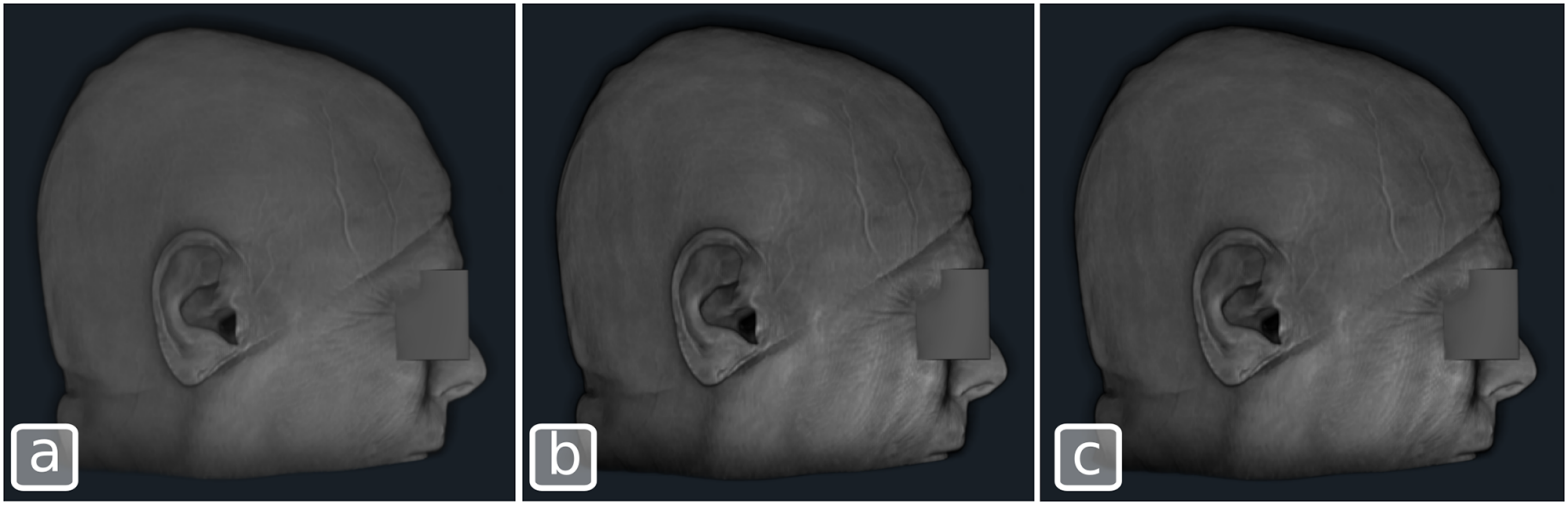
A 320x320x280 voxel volume rendered using the default modes. a) the VTK GPU raycast volume renderer, b) the flexible PRISM GPU raycast framework and c) the PRISM framework with early ray termination (ERT).

Our comparison test ran both renderers on a PC equipped with an Intel Quad Core i7 processor, 32Gb of RAM and an NVidia GeForce GTX 670 graphics card with 4Gb of video memory, running the Ubuntu 14.04 operating system with the proprietary NVidia graphics driver. The test consisted of rendering a single volume of size 320x320x280 voxels into a 1129x1098 pixel window. The volume was rendered 1000 times with each renderer. The resulting frame rates are reported in Table 1. Fig 3 shows the images obtained with both renderers. There is a slight difference of contrast between the images which is most likely due to a window/level adjustment step performed by the VTK renderer and not by PRISM.

**Table 1:** Comparison of the frame rates obtained with the VTK GPU raycast volume renderer and the PRISM framework with and without the early ray termination (ETR) optimization with an alpha threshold of 0.99.

VTK	PRISM	PRISM w. ERT
81.9 fps	78.7 fps	111.9 fps

### Examples of rendering techniques

In this section, we demonstrate the flexibility of the PRISM framework by showing multiple examples of volume rendering methods relevant to medical imaging. Each example is implemented in PRISM with no more than a few lines of custom GLSL code.

One important problem with volume visualization of medical images is the difficulty to produce an image that highlights all details of the anatomical and functional structures of interest while providing sufficient contextual information. We demonstrate the implementation of 3 methods that seek to facilitate separation of focus and context: volume carving[65], opacity peeling[6] and decluttering [66]. Another important area of research in volume rendering is the improvement of depth perception. Several methods that aim to improve depth perception have been presented. In this section, we show 3 examples: Chroma-depth[67], aerial perspective[68] and edge enhancement[69]. Finally, we present a new method where the PRISM framework is used to animate blood flow from a CTA scan that was developed in collaboration with our neurosurgeons.

#### Volume carving

**Fig 4:**
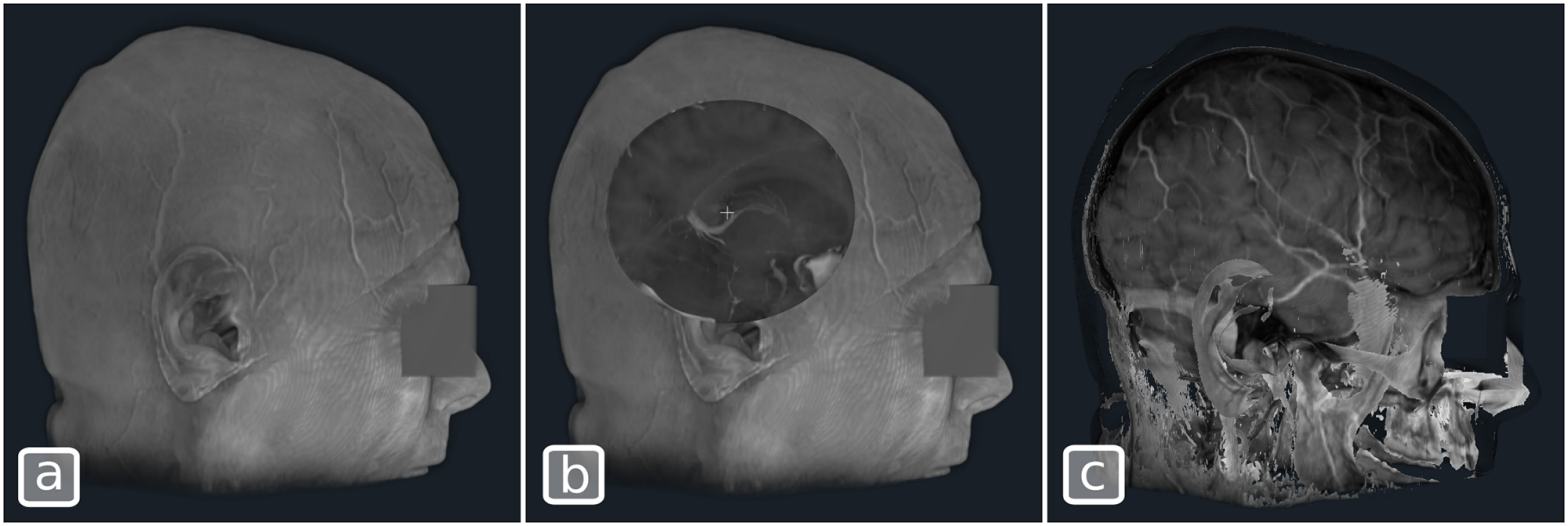
Illustration of volume carving and opacity peeling methods. (a) Original MRI volume, (b) Volume Carving with a spherical tool, (c) Opacity peeling where the first layer is peeled off.

In many visualization problems, structures of interest may be occluded by other regions of the volume with similar intensities. A simple solution to this problem, proposed by Joshi et al. [65], is to interactively carve away a part of the volume. This can be easily done in various ways with PRISM. A simple solution consists in using an **Init** shader that skips the part of the volume that intersects with the area that should be carved away. One simply has to compute the start and stopping coordinates of the carving shape. In our example image in Fig 4b, we apply this technique to carve a spherical region away from an MRI volume. The technique can easily be modified to carve regions of different convex shapes such as a cone or a cylinder. The custom **Init** shader code used is shown in Algorithm 2 below. The interaction point of PRISM is used to drive the center of the spherical region so that it can interactively be manipulated.

**Algorithm 2**. Ray initialization code for volume carving. The code is using PRISM built-in variable **interactionPoint1**, a user-defined 3D coordinate, used here to determine the center of the sphere. **currentDistance** is a built-in variable that specifies the next integration position along the ray. **interactionPoint1** can be manipulated interactively in the 3D window of IBIS.

// Determine if ray is intersecting the sphere

vec3 centerDir = interactionPoint1−rayStart;

float centerProjDist = dot (centerDir, rayDir);

vec3 centerProj = rayStart + rayDir * centerProjDist;

float distCenterProjCenter = length (centerProj−interactionPoint1);

*// if we have an intersection*, *adjust ray integration start*

if (distCenterProjCenter < sphereRadius)

{

 float dcpc2 = distCenterProjCenter * distCenterProjCenter;

 float sr2 = sphereRadius * sphereRadius;

 float intersectDistance = centerProjDist + sqrt (sr2−dcpc2);

 if (intersectDistance > 0.0)

  currentDistance = intersectionDistance;

}

#### Opacity peeling

Another solution to the occlusion problem is the opacity peeling technique proposed by Rezk-Salama and Kolb[6]. It consists in discarding the first *n* layers of tissue when integrating a ray, where *n* is defined by the user. This is done by accumulating alpha along the ray until a certain threshold is reached (T_high). Once the alpha value of a sample falls below a second threshold (T_low), a new layer is started. Ray integration that will contribute to the final image starts only once the user-specified number of layers has been traversed. The **Volume** shader used to generate the image of Fig 4c is shown in Algorithm 3 below.

**Algorithm 3**. Volume shader for the opacity peeling technique. SampleVolumeWithTF is a function built into PRISM to facilitate sampling volume intensity and obtaining the corresponding color in the transfer function. The variables wantedLayer, T_high, T_low are parameters of the algorithm and are defined in an **Init** shader. currentLayer and layerAlpha are working variables also defined in the **Init** shader.

// Sample the volume at the current position

vec4 sample = SampleVolumeWithTF (volIndex, pos);

if (currentLayer < wantedLayer)

{

 *// find if it is time to peel off a layer*

 layerAlpha = layerAlpha + (1.0−layerAlpha) * sample.a;

 if (layerAlpha > T_high && sample.a < T_low)

 {

  currentLayer = currentLayer + 1;

  layerAlpha = 0.0;

 }

}

else

{

 *// peeling done*, *compute regular ray integration*

 sampleRGBA + = sample;

}

#### Decluttering

**Fig 5:**
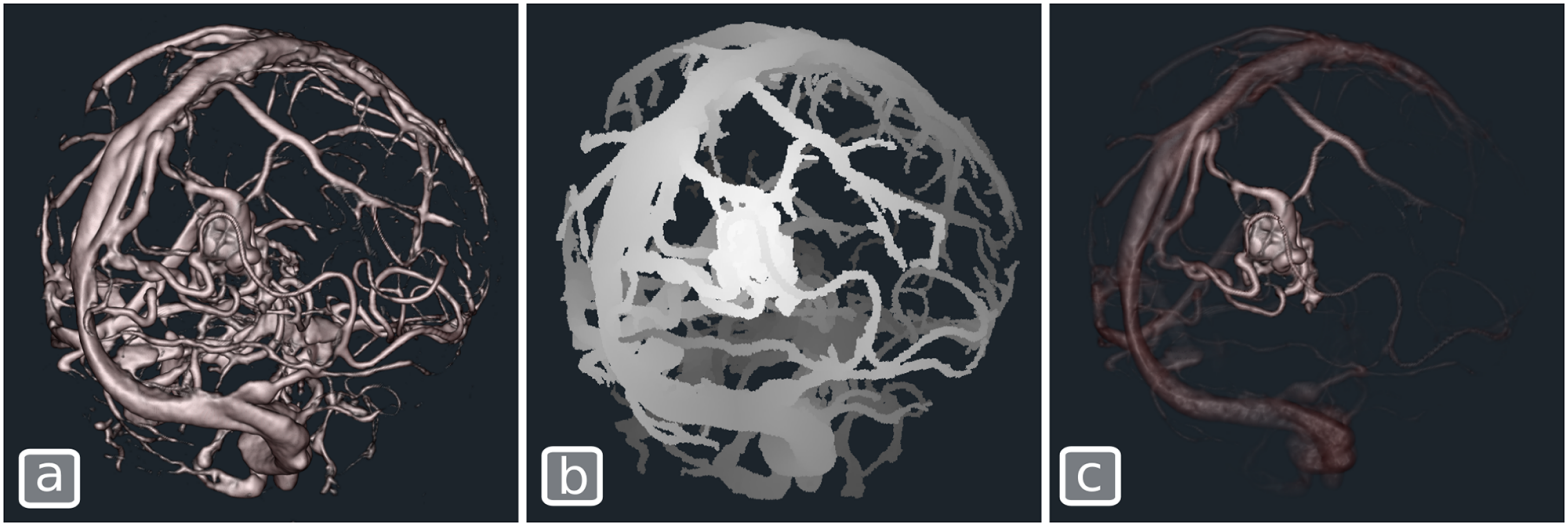
Decluttering an angiographic CT to highlight the structure of interest, an AVM. (a) The original CTA volume rendered with Blinn-Phong shading. (b) A volume containing the topological distance to the AVM for each voxel. (c) The decluttered image obtained by combining (a) and (b).

For many visualization tasks, additional data may be available to help separate structures of interest from the rest of the volume and thus help to focus attention on a particular location or structure. A good example is the visualization of CT angiography (CTA) images where information about blood flow and vessel connectivity can be pre-computed. Raw CTA volumes contain complex vessel structures that often lead to rendered images that are difficult to interpret. We can improve the rendering quality by highlighting specific areas of interest. While a simple geometric or transfer function mechanism may not be able to separate areas of interest, the approach taken in [66] combines the CTA data with a second volume which contains distance, constrained within the vessel tree, between each voxel and the structure of interest, in this case an arteriovenous malformation (AVM). The distance can be computed by a simple level-set front propagation method which is seeded at the AVM nidus [29]. To obtain a decluttered image, the original volume is fed into PRISM as the first input (Fig 5a) and a second volume containing the distance to the AVM (Fig 5b) is also provided. In this example, a very simple two-line **Volume** shader (Algorithm 4) simply multiplies the contribution of this second volume with the previous one, yielding the decluttered image of Fig 5c.

**Algorithm 4**. Volume shader used for the second volume to produce the decluttering effect.

vec4 sample = SampleVolumeWithTF (volIndex, pos);

fullSample * = sample;

Note that in the shader, we don’t use the raw samples from the second volume (distance to the AVM) to modulate color and opacity of the original volume but rather the color-mapped sample (after application of the transfer function). This enables users to interactively adjust the amount of decluttering that is desired by modifying the transfer function.

The simple technique presented here for decluttering is quite general and has various other applications. The second volume can be replaced with any source of complementary information such as a segmentation of a complex region of interest, or other kinds of precomputed topological information.

#### Chroma-depth and aerial perspective

**Fig 6:**
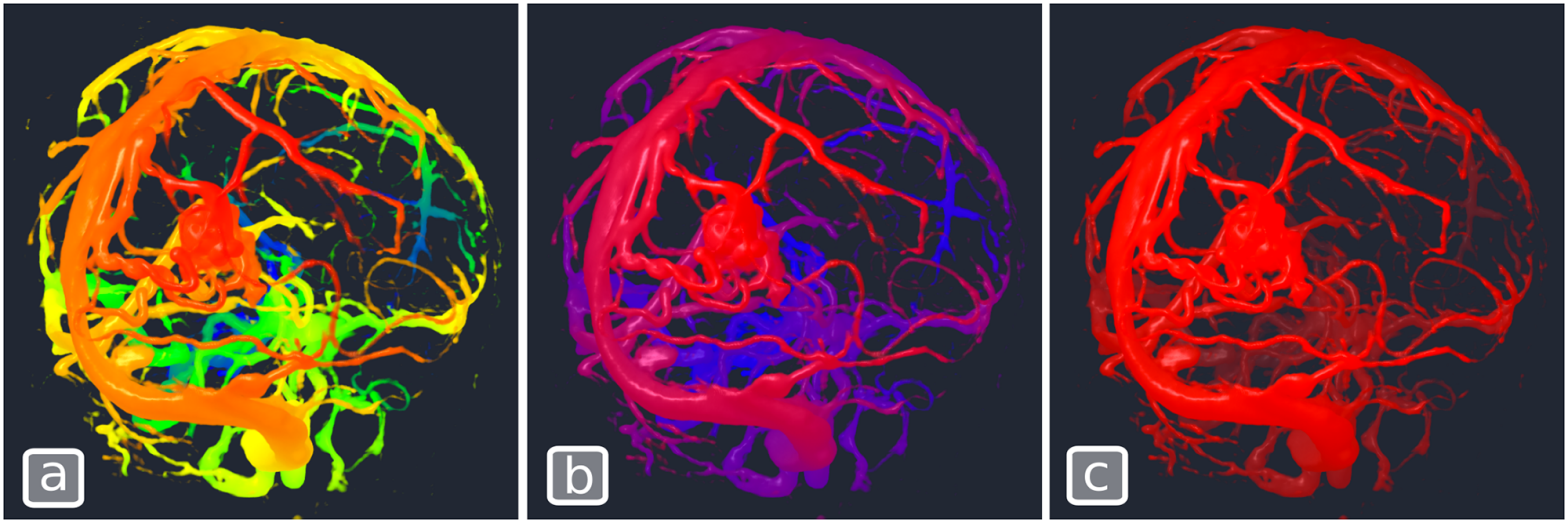
Chroma-depth volume contribution shader used with 3 different color transfer function produces. (a)Chroma-depth, (b)Pseudo chroma-depth and (c)Aerial perspective.

Chroma-depth rendering is a technique initially developed by Richard Steenblik[67] and where depth (distance from the camera) is encoded with color. Chroma-depth and its 2-color variant (pseudo chroma-depth) have been used in various medical image visualization problems [22,23] and have been shown to improve depth perception in the visualization of certain medical image types such as angiographic scans [25]. A similar technique, aerial perspective, represents objects that are more distant as fainter and less contrasted. Both chroma-depth and aerial perspective can be implemented with the same **Volume** shader in PRISM (Algorithm 5). By changing the set of colors used in the transfer function, the same shader code can produce either the chroma-depth (smooth transition between all hues), the pseudo chroma-depth (blue to red gradient) or the aerial perspective effect (background color to white gradient) as illustrated in Fig 6.

**Algorithm 5**. volume contribution custom code for chroma-depth. cameraPosition is a built-in PRISM variable that contains the position of the camera in normalized volume space. volumeDistanceRange is another built-in variable that contains the minimum and maximum distance between the camera and the volume.

// Sample the volume at the current position

vec4 sampleAlpha = SampleVolumeWithTransferFunction (volIndex, pos);

// Compute normalized distance of sample to camera

float distCam = length (pos−cameraPosition);

float range = volumeDistanceRange.y−volumeDistanceRange.x;

float normalizedDistCam = (distCam−volumeDistanceRange.x) / range;

// Use transfer function to determine color based on distance

vec4 sample = SampleTransferFunction (volumeIndex, normalizedDistCam);

// Assign RGB based on distance and alpha based on volume content

sample.a = sampleAlpha.a;

sampleRGBA + = sample;

#### Edge enhancement

Occlusion is an important ordinal depth cue. When visualizing complex structures such as blood vessels in the brain, enhancing edges of the vessels can improve depth perception by disambiguating depth ordering of overlapping vessels. One approach to compute edges in volume rendering is to identify those voxels for which the 3D intensity gradient in the volume data is perpendicular to the viewing direction[69]. In this example, we use this strategy to compute the alpha value in a **Volume** shader using Eq 1 below.

$$\alpha =smoothstep(stepMin,\;stepMax,\;\;||\vec{g}||\;\cdot \;(1-\;\left|\;\vec{g}\;\cdot \;\vec{r}\;\right|))$$ (1)

Where *stepMin* and *stepMax* are user-defined parameters that affect the gradient intensities that are captured by the function, $\vec{g}$ is the finite difference gradient computed by sampling the volume and $\vec{r}$ is a unit vector that defines the direction of the ray being integrated. The PRISM code used to implement this edge rendering technique is shown in Algorithm 6.

**Algorithm 6**. Volume shader used to compute edge enhancement.

// param initialization

float sampleThreshold = 0.1;

float gradStep = 0.0005;

vec2 step = vec2 (0.02, 0.06);

// shading

vec4 sample = SampleVolumeWithTF (volumeIndex, pos);

if (sample.a > sampleThreshold)

{

 vec4 n = ComputeGradient (volumeIndex, pos, gradStep);

 if (n.a > 0.0)

 {

  float factor = n.a * (1.0−abs(dot (rayDir, n.rgb)));

  float alpha = smoothstep (step.x, step.y, factor);

  sampleRGBA = vec4 (1.0, 0.0, 0.0, alpha);

 }

}

**Fig 7:**
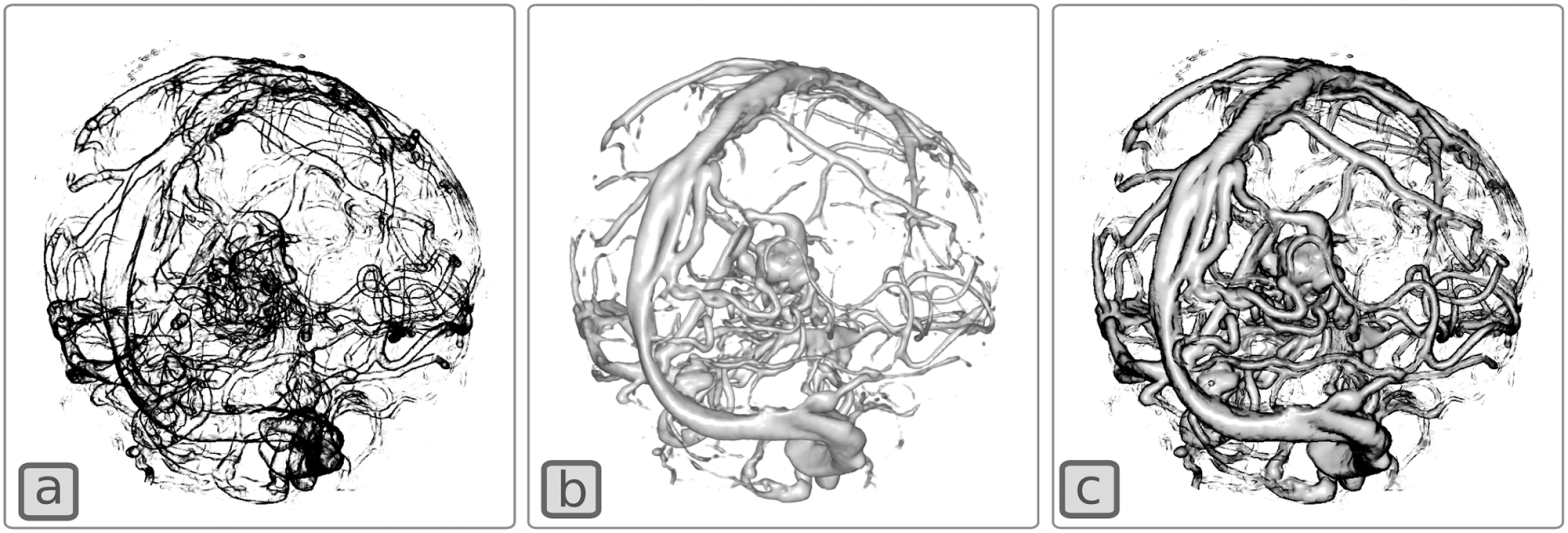
Edge enhancement of an angiographic CT. The volume is rendered with (a) only the edge rendering shader, (b) Only a Blinn-Phong shader, (c) Edge and Blinn-Phong shader combined.

Fig 7 illustrates the effect of the edge shader when applied to an angiographic CT as well as how it can be combined with standard Blinn-Phong shading to produce an edge-enhanced illustration of the blood vessels.

#### Blood flow animation

**Fig 8:**
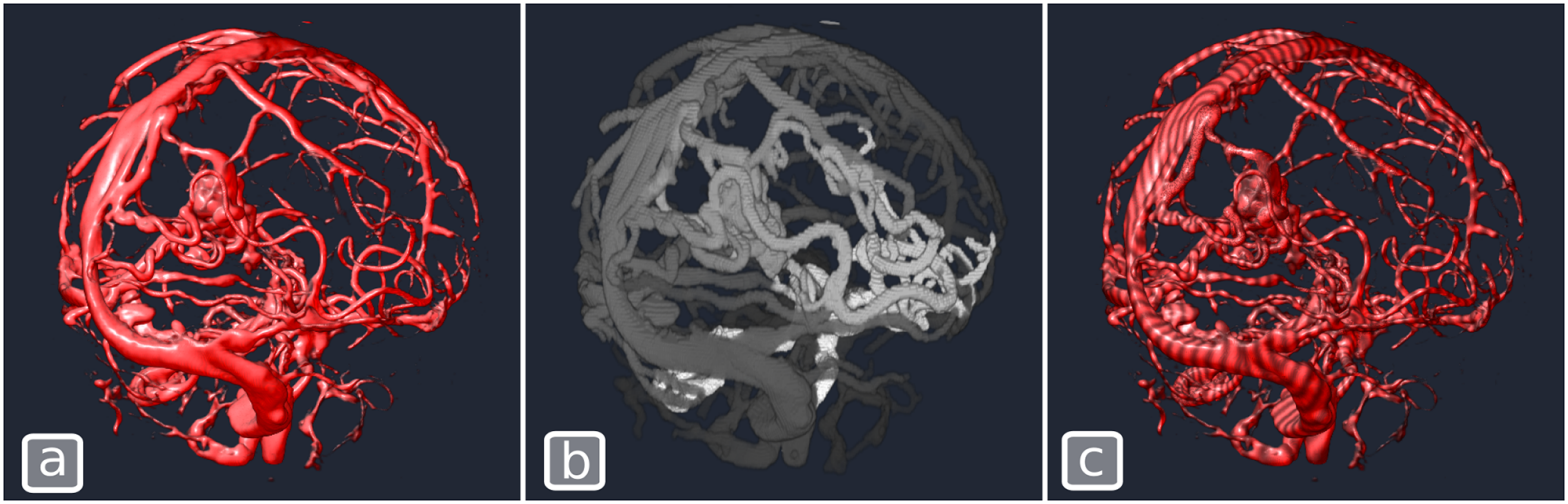
Blood flow depiction. (a) The original CTA volume rendered with Blinn-Phong shading. (b) Volume containing precomputed blood flow information, i.e. distance, within vessels, between entry and exit of blood from the brain. (c) Volume from (b) combined with (a) using the Volume shader of Algorithm 6. When the time parameter is updated to render each frame, the waves move along the vessels in the direction of the blood flow as illustrated in the accompanying video (S1 Video).

In certain situations, it may be helpful to use motion to depict the characteristics of the rendered volume. For example, when rendering angiographic data where the progression of blood in the vessels may be computed using a sequence of images captured at different time points after the injection of a tracing agent. To animate the blood flow, we take advantage of the possibility in PRISM to render multiple volumes simultaneously. The first volume contains a raw angiographic CT and is rendered using a standard Blinn-Phong **Volume** shader. The second volume contains the distance, within vessels, between the entry of blood into the brain (i.e., the carotid and vertebral arteries) and each voxel. In the shader, the values of this second volume are mapped to a sinusoid that is offset as a function of time and the result is used to modulate the color of the first volume. Fig 8 shows a static image produced with this technique. The result can be better appreciated in the video accompanying this paper (S1 Video). Note that the flow information in this example was obtained using a rudimentary simulation and does not depict real blood flow in the brain but is sufficient to demonstrate the visualization principle. The custom **Volume** shader used to render the second volume is shown in Algorithm 7 below.

**Algorithm 7**. Volume shader for the second volume of blood flow animation. SampleVolume is a built-in function of PRISM to obtain the raw volume intensity. time is a built-in variable.

// Sample the volume at the current position

vec4 volumeSample = SampleVolume (volumeIndex, pos);

// Compute a time varying sine wave along vessels

float sineVal = .75 + sin (1000.0 * volumeSample.x + time * float(5.0)) / 4;

// Multiply color by sine wave

sampleRGBA.rgb * = sineVal;

### Examples performance

The performance for all examples above has been measured by rendering 1000 times the final image presented for each of the examples in a VTK window of resolution 1231x1102 pixels. For all examples, the ETR optimization was turned on. The resulting frame rates are shown in Table 2.

**Table 2:** Frame rate in frames per second (fps) for the different volume rendering examples presented in this paper. Note that pseudo chroma-depth and aerial perspective are not listed as they differ from chroma-depth only in the content of the transfer function and not in the computation. In practice the frame rate difference was not measurable.

Volume Carving	102.11 fps
Opacity Peeling	84.85 fps
Decluttering	33.72 fps
Chroma-depth	45.70 fps
Edge enhancement	22.95 fps
Blood flow	49.51 fps

Not surprisingly, volume carving and opacity peeling are the fastest to render as the dataset used in those examples does not have a lot of empty space and thus benefits more from the ETR optimization. Edge enhancement is the slowest. This can probably be explained by the need to compute the gradient in both volume shaders involved (edge-enhancement and Blinn-Phong). Gradient computation requires a large number of volume sampling operations which tends to increase rendering time.

### Usability study

All subjects of the study were easily able to follow the instructions for the first 2 volume rendering examples to reproduce. For the last example, where no explicit instructions were provided, 3 out of 5 participants were able to reproduce the image presented to them by employing the same strategy as the one presented in the section on Decluttering above. A fourth candidate was able to produce an image that was visually close to the target image, but using a different combination of volumes and shaders. Thus, for most participants, a 5 minutes tutorial and the execution of 2 examples was enough to be able to compose a new example on their own using a set of shaders already developed by experts.

We analyzed the video recorded during the experiment with the 5 subjects to find the main usability issues of the system. The main complaint from the users concerned the manipulation of the transfer function, a feature that is not specific to PRISM, but almost universal in volume rendering systems. All subjects reported a difficulty to understand the relationship between the transfer function and the rendered result. Another request frequently made by users was to provide a basic documentation that describes the behavior of the existing shaders. Finally, two GUI adjustments were unanimously suggested by all subjects: Clearly distinguish buttons to add and remove volumes from the buttons to add and remove shaders and provide tooltips for all the GUI element.

The results from the online questionnaire filled by participants are summarized in Table 3 below.

**Table 3:** Mean and standard deviation of the answers provided by the users on the online questionnaire. Each question is answered on a scale of 1 to 5 (1 = strongly disagree, 5 = strongly agree). The System Usability Scale (SUS) score (last line of part 2) is obtained by subtracting 1 from the score of each odd-numbered question and subtracting the score of even-numbered from 5. We sum the results obtained for each question and multiply by 2.5 to obtain the final score.

**Part 1: experience with volume rendering**	
1- I have often used volume rendering software before	1.4 ± 0.9
2- I have a deep understanding of the underlying principles of volume rendering	1.8 ± 1.1
	
**Part 2: System Usability Scale**	
1—I think that I would like to use PRISM	3.8 ± 1.1
2—I found PRISM unnecessarily complex	2.0 ± 0.7
3—I thought that PRISM was easy to use	3.2 ± 0.8
4—I think that I would need the support of a technical person to be able to use PRISM	2.4 ± 1.5
5—I found that the various functions in PRISM were well integrated	4.2 ± 1.3
6—I thought there was too much inconsistency in PRISM	1.4 ± 0.5
7—I would imagine that most people would learn to use PRISM very quickly	3.8 ± 1.3
8—I found PRISM very cumbersome to use	1.6 ± 0.5
9—I felt very confident using PRISM	3.0 ± 1.4
10—I needed to learn a lot of things before I could get going with PRISM	2.4 ± 1.5
**System Usability Scale (SUS)** score (/100)	**70.5 ± 4.5**
	
**Part 3: PRISM specific questions**	
1—It was easy to combine multiple volumes using PRISM	4.8 ± 0.4
2—It was easy to understand the influence of each type of shader on the rendered image	3.4 ± 1.1
3—The system would be appropriate for medical technicians and doctors	4.2 ± 0.8
4—The system would be appropriate for medical imaging specialists	4.8 ± 0.4
5—The system would be appropriate for computer graphics programmers	4.4 ± 0.5
	

## Discussion

In this paper, we demonstrate both the flexibility of the PRISM framework and the quality of its implementation. The tests of the PRISM framework show that its performance and the default mode rendering quality is equivalent to that of a widely used open source GPU ray-casting module provided by VTK. For most other algorithms presented, no reference implementation is available to compare with. However, given the public availability of both the code and complete example files for all methods we presented in this paper, it is easy for other developers to propose alternative implementations and compare their performance with PRISM.

Through several examples, we have shown that the PRISM framework can be used to implement a wide variety of volume rendering methods. In many cases, the ray casting approach of the framework simplifies the implementation of new methods due to its simplicity and allows for powerful optimization techniques such as early ray termination and empty space skipping.

The results of the usability study that was conducted with medical imaging experts with little or no experience of volume rendering shows that the system allows this category of users to produce various types of rendering that are not available with conventional volume renderers. They can do so by combining several shader components available from a public database using a simple graphical user interface and without having to program.

The SUS score of 70.5 places the usability of the system in the range of acceptability according to Bangor et al. who analyzed the result of more than 200 studies involving more than 2300 participants [70]. It is difficult to draw a clear conclusion from the score of individual questions, but we note that the questions that had the most negative impact on the final SUS score (3 and 9) suggest that the score might be improved with a little more training of the subjects. The answers to the first 2 questions of part 3 of the questionnaire suggest that the difficulties experimented by the users are related to the manipulation of preexisting shaders more than to the combination of multiple volumes. Results could probably be improved if, as suggested by the users in their comments, documentation was provided for each one of them. The answers to the last three questions suggests that the PRISM system is more appropriate for medical imaging specialists than for other categories of users. Considering that all the participants to the study fall into this category, they may not be able to judge the utility of the system for medical technicians and doctors as accurately. The usability of the system for graphics programmers has been extensively tested through its regular use at our institute. However, it still needs to be tested with medical personnel involved with imaging. Given the challenges encountered by medical imaging specialists, especially with the concept of the transfer function, another layer of abstraction may be needed for this category of users. This new level of abstraction would allow programmers and medical imaging specialists to package a specific combination of volumes, shader components and transfer functions such that medical personnel would only need to adjust a very small number of high-level parameters in order to produce the desired image.

This work is not without some disadvantages. In particular, the proposed ray casting approach is implemented in a single pass in the GPU, making it impossible for the ray integration code to access information computed for neighboring pixels. This disadvantage can often be compensated by precomputing volumetric information or integrating each ray over a larger neighborhood, but both alternatives are computationally expensive. In contrast, slice-based approaches[43] conceptually propagate all rays in parallel, with a new rendering pass for every step. It is thus possible to access neighborhood information computed in previous steps. This property facilitates the development of methods such as shadow and scattering[11], depth of focus[21] or volumetric halos [24]. In the future, a new hybrid approach may be necessary to combine the advantages of both slice-based and ray casting volume rendering.

## Future work

One of the current limitations of PRISM is the requirement that all volumes rendered simultaneously share the same bounding box. In the future, we will implement support for non-aligned volumes based on the solution proposed by Bozorgi et al. [47]. Another simple feature to add is support for multi-dimensional transfer functions. A wide variety of publications employ this strategy to simplify separation of relevant tissues in volumetric data. Finally, in a future version of PRISM, we will allow shaders to define custom parameters that will be exposed to the client program. This will enable automatic generation of GUI elements in the client programs to adjust those parameters and allow non-programmers to experiment more easily with different combinations.

## Conclusion

In this paper, we have presented an easy-to-program open-source ray casting volume renderer and have shown that it can be used to quickly implement a variety of techniques appropriate for specific visualization problems. Furthermore, we have shown that a simple graphical user interface allows non-expert users to experiment with a list of already existing shaders to find the one that best suits the visualization task at hand. We believe that the PRISM framework has the potential to greatly simplify sharing of volume rendering algorithms in the medical imaging research community and thus, to accelerate the pace of research in this field.

## Supporting information

S1 Appendix PRISM built-in functions and variables. (PDF)

S2 Appendix Instructions—PRISM usability study. (PDF)

S3 Appendix Usability study questionnaire. (PDF)

S1 Video PRISM demonstration video. (MP4)
