# Supplementary material for: PRISM: An open source framework for the interactive design of GPU volume rendering shaders
Source: PLoS One. 2018 Mar 13;13(3):e0193636. doi: 10.1371/journal.pone.0193636 (PMC5849289; doi:10.1371/journal.pone.0193636)
Supplement: S1 Appendix — (PDF) [file pone.0193636.s001.pdf]

S1 Appendix – PRISM built-in functions and variables

Variables

Variable	Type	Description
**rayStart**	vec3	Starting point of the ray
**rayDir**	vec3	Direction of the ray (unit vector)
**pos**	vec3	Current position along the ray
**currentDistance**	float	Current distance from the start of the ray
**volumeIndex**	int	Id of the volume associated with the current shader (volume shaders only)
**sampleRGBA**	vec4	The output of Volume shaders. This represents the combined contribution of all active Volume shaders.
**cameraPosition**	vec3	Position of the VTK camera
**volumeDistanceRange**	vec2	Distances of the points of the volumes closer and further from the camera.
**interactionPoint1**	vec3	Position of a user defined point that may be used for interaction
**interactionPoint2**	vec3	Position of a user defined point that may be used for interaction

Note: all positions, orientations and distance variables are relative to normalized volume coordinates (openGL 3D texture coordinates).

Functions

Function	Return Type	Description
**SampleVolume(int index, vec3 pos)**	vec4	Sample volume *index* at position *pos*.
**SampleVolumeWithTF(int index, vec3 pos)**	vec4	Sample volume *index* at position *pos* and color map the result using the transfer function associated with the volume
**SampleTF(int index, float value)**	vec4	Samples transfer function associated with volume *index*.
**ComputeGradient(int index, vec3 pos, float radius)**	vec4	Compute finite difference gradient at position pos in volume index. Radius is the size of the region considered.

	The 4th component of the return value contains the gradient amplitude.
