# Supplementary material for: PRISM: An open source framework for the interactive design of GPU volume rendering shaders
Source: PLoS One. 2018 Mar 13;13(3):e0193636. doi: 10.1371/journal.pone.0193636 (PMC5849289; doi:10.1371/journal.pone.0193636)
Supplement: S2 Appendix — (PDF) [file pone.0193636.s002.pdf]

Instructions - PRISM usability study

Example 1 - Volume Carving

1. Adjust transfer function to match the figure
2. Set the Ray Init shader to “**carve-sphere**”
3. Show interaction widget
4. Move point and view around to see the effect of the shader

Example 2 - Blood flow

1. Adjust transfer function to match more or less the example figure
2. Set the shader on “**Volume 0**” to “**phong shading**”
3. Press the add volume (**+**) button.
4. In the “**Volume 1**” section, choose volume “**3_ArteryFlow.mnc**”
5. In the “**Volume 1**” section, choose shader “**sine-wave**”
6. In the “**Volume 1**” section, check the “16 bits” checkbox
7. Check the “**Animate**” checkbox

Example 3 - Decluttering

By default, the system is showing only the basic anatomical volume.

1. Visualize the 2 loaded volumes individually to understand what they contain.
2. Comment on the content of the volumes and the example figure to reproduce.
3. Try to reproduce the rendering of the example figure by combining the volumes with the appropriate shaders.

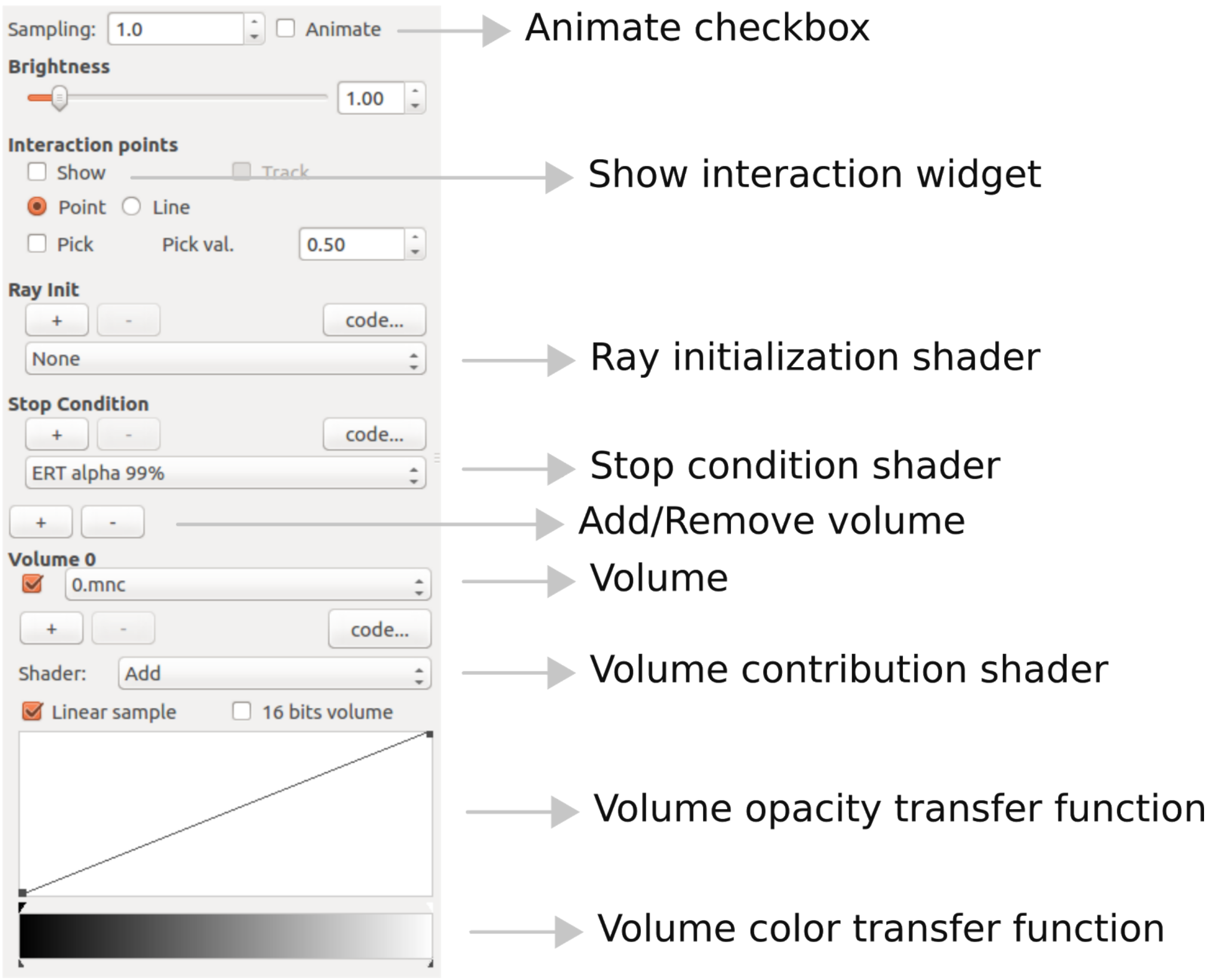

Sampling: 1.0
Animate
Brightness
1.00
Interaction points
Show
Track
Point
Line
Pick
Pick val.
0.50
Ray Init
code...
None
Stop Condition
code...
ERT alpha 99%
Volume 0
0.mnc
code...
Shader:
Add
Linear sample
16 bits volume
Animate checkbox
Show interaction widget
Ray initialization shader
Stop condition shader
Add/Remove volume
Volume
Volume contribution shader
Volume opacity transfer function
Volume color transfer function
