# Supplementary material for: PRISM: An open source framework for the interactive design of GPU volume rendering shaders
Source: PLoS One. 2018 Mar 13;13(3):e0193636. doi: 10.1371/journal.pone.0193636 (PMC5849289; doi:10.1371/journal.pone.0193636)
Supplement: S3 Appendix — (PDF) [file pone.0193636.s003.pdf]

PRISM Volume renderer usability study

The questionnaire is divided into 3 parts. First, 2 questions about prior experience of the participant with volume rendering. Part 2 is a standard System Usability Scale (SUS) test. Finally, part 3 contains questions more specific to the functionality of the PRISM framework. All questions except for the last one should be answered on a 1 to 5 scale (1 = strongly disagree, 5 = strongly agree)

*Required

1. **I have often used volume rendering software before** *

   Mark only one oval.

	1	2	3	4	5	
Strongly disagree	⬭	⬭	⬭	⬭	⬭	Strongly agree

2. **I have a deep understanding of the underlying principles of volume rendering** *

   Mark only one oval.

	1	2	3	4	5	
Strongly disagree	⬭	⬭	⬭	⬭	⬭	Strongly agree

System Usability Scale

3. **I think that I would like to use PRISM** *

   Mark only one oval.

	1	2	3	4	5	
Strongly disagree	⬭	⬭	⬭	⬭	⬭	Strongly agree

4. **I found PRISM unnecessarily complex** *

   Mark only one oval.

	1	2	3	4	5	
Strongly disagree	⬭	⬭	⬭	⬭	⬭	Strongly agree

5. **I thought that PRISM was easy to use** *

   Mark only one oval.

	1	2	3	4	5	
Strongly disagree	⬭	⬭	⬭	⬭	⬭	Strongly agree

6. **I think that I would need the support of a technical person to be able to use PRISM** *

   Mark only one oval.

	1	2	3	4	5	
Strongly disagree	⬭	⬭	⬭	⬭	⬭	Strongly agree

7. **I found that the various functions in PRISM were well integrated** *
Mark only one oval.

	1	2	3	4	5	
Strongly disagree	◯	◯	◯	◯	◯	Strongly agree

8. **I thought there was too much inconsistency in PRISM** *
Mark only one oval.

	1	2	3	4	5	
Strongly disagree	◯	◯	◯	◯	◯	Strongly agree

9. **I would imagine that most people would learn to use PRISM very quickly** *
Mark only one oval.

	1	2	3	4	5	
Strongly disagree	◯	◯	◯	◯	◯	Strongly agree

10. **I found PRISM very cumbersome to use** *
Mark only one oval.

	1	2	3	4	5	
Strongly disagree	◯	◯	◯	◯	◯	Strongly agree

11. **I felt very confident using PRISM** *
Mark only one oval.

	1	2	3	4	5	
Strongly disagree	◯	◯	◯	◯	◯	Strongly agree

12. **I needed to learn a lot of things before I could get going with PRISM** *
Mark only one oval.

	1	2	3	4	5	
Strongly disagree	◯	◯	◯	◯	◯	Strongly agree

PRISM specific questions

13. **It was easy to combine multiple volumes using PRISM** *
Mark only one oval.

	1	2	3	4	5	
Strongly disagree	◯	◯	◯	◯	◯	Strongly agree

14. **It was easy to understand the influence of each type of shader on the rendered image** *
Mark only one oval.

	1	2	3	4	5	
Strongly disagree	◯	◯	◯	◯	◯	Strongly agree

15. **The system would be appropriate for medical technicians and doctors** *
Mark only one oval.

	1	2	3	4	5	
Strongly disagree	◯	◯	◯	◯	◯	Strongly agree

16. **The system would be appropriate for medical imaging specialists** *
Mark only one oval.

	1	2	3	4	5	
Strongly disagree	◯	◯	◯	◯	◯	Strongly agree

17. **The system would be appropriate for computer graphics programmers** *
Mark only one oval.

	1	2	3	4	5	
Strongly disagree	◯	◯	◯	◯	◯	Strongly agree

18. **Can you suggest any improvement to the PRISM framework that would facilitate the creation of complex visualization by non-programmers?**

Powered by
Google Forms
